# Metabolic characterisation of THP-1 macrophage polarisation using LC–MS-based metabolite profiling

**DOI:** 10.1007/s11306-020-01656-4

**Published:** 2020-02-29

**Authors:** Alaa Abuawad, Chidimma Mbadugha, Amir M. Ghaemmaghami, Dong-Hyun Kim

**Affiliations:** 1 Division of Advanced Materials and Healthcare Technologies, Centre for Analytical Bioscience, School of Pharmacy, University of Nottingham, Nottingham, UK; 2 Department of Pharmaceutical Sciences and Pharmaceutics, Faculty of Pharmacy, Applied Science Private University, Amman, Jordan; 3 Division of Immunology, School of Life Sciences, Faculty of Medicine and Health Sciences, University of Nottingham, Nottingham, UK

**Keywords:** LC–MS, Macrophage polarisation, THP-1 cell, Metabolite profiling, Metabolic pathway analysis

## Abstract

**Introduction:**

Macrophages constitute a heterogeneous population of functionally distinct cells involved in several physiological and pathological processes. They display remarkable plasticity by changing their phenotype and function in response to environmental cues representing a spectrum of different functional phenotypes. The so-called M1 and M2 macrophages are often considered as representative of pro- and anti-inflammatory ends of such spectrum. Metabolomics approach is a powerful tool providing important chemical information about the cellular phenotype of living systems, and the changes in their metabolic pathways in response to various perturbations.

**Objectives:**

This study aimed to characterise M1 and M2 phenotypes in THP-1 macrophages in order to identify characteristic metabolites of each polarisation state.

**Methods:**

Herein, untargeted liquid chromatography (LC)–mass spectrometry (MS)-based metabolite profiling was applied to characterise the metabolic profile of M1-like and M2-like THP-1 macrophages.

**Results:**

The results showed that M1 and M2 macrophages have distinct metabolic profiles. Sphingolipid and pyrimidine metabolism was significantly changed in M1 macrophages whereas arginine, proline, alanine, aspartate and glutamate metabolism was significantly altered in M2 macrophages.

**Conclusion:**

This study represents successful application of LC–MS metabolomics approach to characterise M1 and M2 macrophages providing functional readouts that show unique metabolic signature for each phenotype. These data could contribute to a better understanding of M1 and M2 functional properties and could pave the way for developing new therapeutics targeting different immune diseases.

**Electronic supplementary material:**

The online version of this article (10.1007/s11306-020-01656-4) contains supplementary material, which is available to authorized users.

## Introduction

Macrophages as effector cells in the immune system, play a critical role in innate and adaptive immunity (Martinez et al. 2008). They are a heterogeneous population of cells with high degree of plasticity and ability to acquire distinct functional phenotypes in response to different environmental changes and stimuli such as microbial, parasitic, and viral antigens, or apoptotic cells (Vogel et al. 2014). Many macrophage populations have now been identified that display a broad spectrum of functional phenotypes (Mantovani et al. 2005). However, two major populations of macrophages with distinct functions represent the extreme polarisation states of macrophages; pro-inflammatory M1 macrophages and anti-inflammatory M2 macrophages (Filardy et al. 2010). M1 macrophages play a crucial role against infectious microbes (e.g. bacteria and viruses) and tumour progression whereas M2 macrophages are involved in tissue repair, wound healing, and in response against parasitic infections (Chanput et al. 2013).

Resting or naïve macrophages (M0) can be polarised to a pro-inflammatory M1 phenotype by exposure to granulocyte macrophage colony-stimulating factor (GM-CSF), Th1 cytokines like IFN-γ and TNF-α, and other toll-like receptor (TLR) ligands such as bacterial lipopolysaccharide (LPS). Polarisation towards anti-inflammatory M2 phenotype, on the other hand, is primarily induced via exposure to macrophage colony-stimulating factor (M-CSF) and Th2 cytokines like IL-4 or IL-13 (Martinez et al. 2008; Vogel et al. 2014). The balance between pro- and anti-inflammatory macrophage phenotypes plays a central role in maintenance of tissue homeostasis under steady-state conditions and after exposure to pathogens and tissue damage (Gu et al. 2017; Mandal et al. 2011). This is exemplified by various autoimmune [e.g. rheumatoid arthritis (Gu et al. 2017), multiple sclerosis (Bruck et al. 1996) and Crohn’s disease (Smith et al. 2009)] and chronic inflammatory diseases [e.g. alcoholic and non-alcoholic liver diseases (Tilg and Diehl 2000), atherosclerosis (Viola and Soehnlein 2015), and diabetes (Kraakman et al. 2014)] where dysregulation in macrophage function is thought to play a central role (Mandal et al. 2011; Murphy et al. 2003; Smith et al. 2009). However, the molecular events that control M1 and M2 polarisation and underpin different functions of pro- and anti-inflammatory macrophages are still poorly understood (Lopez-Castejon et al. 2011).

Characterisation of macrophage polarisation has been studied widely. Chanput et al. recently reported characterisation of THP-1 macrophages for both M1 and M2 phenotypes using expression of chemokines and surface marker receptors. This study demonstrated that chemokines and surface receptors could be inappropriate markers for characterising different macrophage polarising states particularly for M2 subset which was less sharply defined because chemokines are affected by secondary cell–cell signals that are crucial to drive M2 polarisation (Chanput et al. 2013). In another study, Lopez-Castejon et al. have shown specific gene expression profile which controls M1 and M2 macrophage phenotypes (Lopez-Castejon et al. 2011). Also, Sierra-Filardi et al. demonstrated a characteristic high expression of C–C chemokine receptor type 2 (CCR2) and C–C chemokine ligand 2 (CCL2) genes in M1 and M2 macrophages, respectively (Sierra-Filardi et al. 2014). However, gene expression profiling does not account for differences in functional phenotype and cannot reveal the complexity of biological samples (Kosmides et al. 2013). Furthermore, a great overlap in the expression of surface markers has been reported between different macrophage subsets and more importantly deciphering function based on surface phenotype is not always possible (Geissmann et al. 2010). Therefore, there is a need for more functional biological readouts that not only allow better classification of different macrophage subsets but also provide better insight into the molecular mechanisms that underpin distinct functional and phenotypical subsets. Given the mounting evidence highlighting a strong correlation between the metabolic state and the functional phenotype of macrophages, metabolite profiling could prove as a powerful tool for better classification and understanding of different macrophage phenotypes (Galván-Peña and O’Neill 2014; Rath et al. 2014). For examples, M1 macrophages have been characterised by high levels of glycolysis and flux through the pentose phosphate pathway (PPP), and an increase in particular tricarboxylic cycle (TCA cycle) metabolites such as citrate, succinate and itaconate. Moreover, M1 macrophages have increased fatty acids synthesis (Galván-Peña and O’Neill 2014; Jha et al. 2015). On the other hand, M2 macrophages adopt oxidative phosphorylation and fatty acids oxidation (Covarrubias et al. 2015). These key metabolic differences between M1 and M2 macrophages are widely accepted. However, the metabolic mechanisms that underpin the orchestrating of their profiles remain not well understood. Also, M1 macrophages are distinctive with the expression of nitric oxide synthase (iNOS) that facilitates the production of NO, which has anti-microbial activity and cytotoxicity whereas M2 macrophages express arginase-1 instead that is required in the production of polyamines which are necessary for cell proliferation, collagen synthesis and tissue remodelling (Covarrubias et al. 2015; Orihuela et al. 2016).

Liquid chromatography (LC)–mass spectrometry (MS)-based metabolomics approach is a powerful tool for quantifying metabolites as well as for identifying known and unknown compounds in biological samples (Surrati et al. 2016). Therefore, this technique has been used widely for disease diagnostic biomarker discovery (Jansson et al. 2009) as well as evaluation of drug toxicity (Sun et al. 2009). In addition, LC–MS-based approach has been employed to investigate the function of immune cells and immune responses by monitoring level changes of signalling molecules. Tannahill et al. have successfully revealed succinate as an inflammatory signal in lipopolysaccharide (LPS)-activated bone marrow-derived macrophages (BMDMs) using LC–MS metabolomics approach (Tannahill et al. 2013a). Also, Hollenbaugh et al. have demonstrated that LC–MS/MS metabolomics was an effective approach to monitor various metabolic alterations in HIV-1 infected primary human CD4 + T cells and a macrophage model system (Hollenbaugh et al. 2011). Moreover, LC–MS metabolomics has been effectively used to monitor LPS-induced changes in amino acid metabolism of macrophage-like cell line (RAW 264.7) and to identify relevant pathways (Suh et al. 2012). In the context of macrophages polarisation, a recent study has used integrated transcriptomics and LC–MS-based metabolomics to investigate the polarisation and the distinct metabolic profiles of M1 and M2 macrophages in mice. They demonstrated that M2 macrophages activate glutamine catabolism and UDP-*N*-acetylglucosamine (UDP-GlcNAc)-related modules. On the other hand, they identified interruption in the TCA cycle between isocitrate and α-ketoglutarate (OXPHOS) in M1 macrophages (Jha et al. 2015). Using LC–MS metabolomics, Mills et al. have shown that itaconate is an anti-inflammatory metabolites which has important role in regulation of macrophage functions (Mills et al. 2018). However, Rattigan et al. demonstrated a complex role of itaconate as an inflammatory and anti-inflammatory key metabolite (Rattigan et al. 2018). Moreover, another metabolomics study of macrophages has identified succinate as a participant in innate immune signalling in response to LPS (M1 macrophages) where its increase results from impaired TCA cycle and leads to boost the production of the inflammatory cytokine, IL-1β during inflammation (Tannahill et al. 2013b). Also, Freemerman et al. demonstrated that driving glucose uptake and metabolism through glucose transporters in macrophages induces a proinflammatory response and the metabolomics analysis revealed increase in glycolysis, PPP, purine and pyrimidine metabolism (Freemerman et al. 2014). However, metabolic signature of human macrophage polarisation states has not been investigated yet.

In this study, untargeted LC–MS-based metabolite profiling was employed to characterise M1 and M2 phenotypes in THP-1 macrophages in order to identify the key characteristic metabolites of each polarisation state in these cells. Our data clearly showed distinct metabolic signature and associated changes of relevant metabolites in M1 and M2 macrophages which could not only provide better understanding of the functional properties of different macrophage subsets but also pave the way for identifying new key characteristic metabolites and potentially therapeutic targets in different inflammatory diseases.

## Materials and methods

### Materials

THP-1 cell line was purchased from ATCC, USA. RPMI 1640 medium, phosphate buffered saline (PBS) and *E. coli* lipopolysaccharide were purchased from Sigma-Aldrich, UK. GM-CSF, M-CSF and IL-4 were purchased from Miltenyi Biotec, Germany. IFN-γ was purchased from R&D Systems, USA. Methanol and acetonitrile were purchased from Fisher Scientific, UK. All solvents were LC–MS grade.

### Methods

#### Cell culture

Human THP-1 cells were cultured and differentiated as previously described in (Chanput et al. 2013). Briefly, THP-1 cells were grown in T75 tissue culture flasks using RPMI 1640 supplemented with 10% heat-inactivated fetal bovine serum (FBS), 1% l-glutamine, and 1% penicillin–streptomycin. Cells were then incubated at 37 °C and 5% CO_2_.

#### Differentiation and polarisation of THP-1 cells

THP-1 cells were differentiated into naïve macrophages-like state (M0-like) by treating with phorbol-12-myristate-13-acetate (PMA). Six million cells were seeded per flask (T25 tissue culture flask), and then the cells were treated with PMA containing media with a final concentration of 50 ng/ml of PMA and incubated for 6 h. After the incubation period, the cells were further treated with either 50 ng/ml GM-CSF, 100 ng/ml LPS and 20 ng/ml IFN-γ or 50 ng/ml M-CSF and 20 ng/ml IL-4 for up to 72 h to generate M1 or M2 polarised cells, respectively (Caras et al. 2011). This polarisation protocol was adapted from previous studies (Caras et al. 2011; Rostam et al. 2017) and the cell phenotypes were confirmed by conventional assays (Supplementary 1). Cells treated with PMA only were used as controls (M0). Six replicates for each condition were prepared.

#### Sample preparation and metabolite extraction

After incubated for 72 h, the media containing PMA and polarising agents were removed. The cells were then washed once with pre-warmed PBS (37 °C) and 500 µl of pre-cooled methanol at − 48 °C was used for metabolism quenching and metabolite extraction. The cells were harvested using a plastic scraper whilst being kept on ice and the extracts were transferred into pre-cooled fresh tubes (4 °C). The cell extract was vortexed for 1 h and centrifuged at 16,100×*g* for 10 min at 4 °C. After the centrifugation, the supernatants were transferred into pre-cooled fresh tubes (4 °C) and they were then dried under vacuum and reconstituted in 70 µl of methanol. The samples were stored at − 80 °C prior to LC–MS analysis. To assess the instrument performance, a quality control (QC) sample was prepared by mixing equal volume of each sample.

#### Analytical methodologies

LC–MS-based metabolite profiling was performed on an Accela system coupled to an Exactive MS (Thermo Fisher Scientific, Hemel Hempstead, UK) operating with electrospray ionisation (ESI) running in the negative (ESI−) and positive (ESI+) modes as previously described in (Kim et al. 2015). Briefly, the spray voltage was 4500 V (ESI+) and 3500 V (ESI−), capillary voltage was 40 V (ESI+) and 30 V (ESI−), tube lens voltage was 70 V for the both modes and skimmer voltage was 20 V (ESI+) and 18 V (ESI−). The temperature for capillary and probe was maintained at 275 °C and 150 °C, respectively. Chromatographic separation was carried out using ZIC-pHILIC (4.6 × 150 mm and 5 μm particle size, Merck Sequant). The mobile phase composed of 20 mM ammonium carbonate in water (solvent A) and 100% acetonitrile (solvent B). Metabolites were separated according to a linear gradient as following: 0–15 min (20% A), 15–17 min (95% A), and 17–24 min (20% A) at 300 µl/min flow rate. The injection volume of 10 µl and the column was kept at 45 °C.

#### Data processing and metabolites identification

To process raw data obtained from LC–MS, XCMS and mzMatch were used for untargeted peak-picking and peak matching, respectively (Scheltema et al. 2011; Tautenhahn et al. 2008). IDEOM was employed for putative metabolite identification and noise filtering with default parameters (Creek et al. 2012). Briefly, retention time (RT) for identification of authentic standards was 5%, RT for identification for calculated RT was 50%, mass accuracy for mass identification was 3 ppm. Metabolites were identified with four levels of confidence; level 1 (L1) identification was based on matching the accurate masses, MS/MS fragmentation and retention times of the detected metabolite peaks with those of 250 authentic standards which were co-analysed with the samples under identical experimental conditions, level 2 (L2) identification was based on matching the accurate masses and retention times (two orthogonal data) of the detected metabolite peaks with those of the authentic standards, level 3 (L3) identification was carried out when the predicted retention times were employed due to the lack of standards and level 4 (L4) identification was based on unambiguously assigned molecular formulas but insufficient evidence exists to propose possible structures. The identification criteria were according to the metabolomics standards initiative and scale by (Schymanski et al. 2014; Sumner et al. 2014, 2007).

Pre-processed data were analysed by performing multivariate and univariate analysis. Orthogonal partial least squares-discriminant analysis (OPLS-DA) was carried out by SIMCA-P v13.0.2 (Umetrics, Umea, Sweden) as a supervised multivariate model. This multivariate analysis was used as the first step for visualising data with sample classes and evaluating the metabolome changes in M0, M1, and M2 groups. The key mass ions representing potential characteristic metabolites were determined based on variable importance in projection (VIP) values obtained from two-way orthogonal comparisons. Mass ions with VIP values greater than one were considered as discriminant key characteristic metabolites. Univariate analysis was also performed in parallel to multivariate analysis to identify significant mass ions. T-test with false discovery rate (FDR) correction was performed using MetaboAnalyst (Leon et al. 2013) to determine the significantly changed mass ions between unpolarised THP-1 macrophages (M0) and polarised groups (M1 and M2). MetaboAnalayst and Kyoto Encyclopedia of Genes and Genomes (KEGG) database were used to analyse and visualise the affected pathway.

## Results and discussion

### Macrophages characterisation using surface markers and cytokine profile

**Fig. 1:**
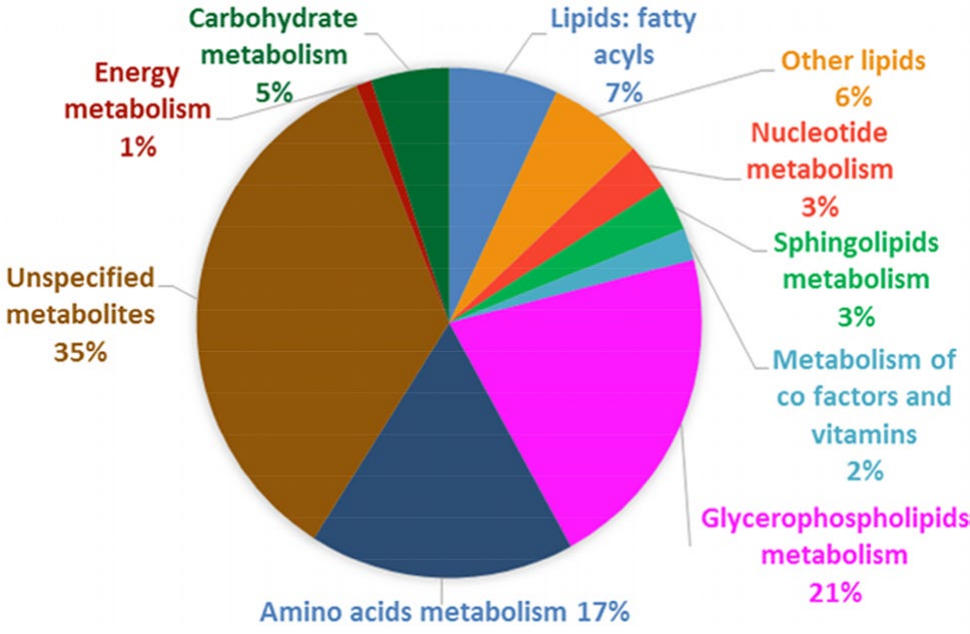
Functional categories of the intracellular metabolites produced by THP-1 macrophages. In total, 644 metabolites were putatively identified in THP-1 macrophage extracts using LC–MS-based metabolite profiling

**Fig. 2:**
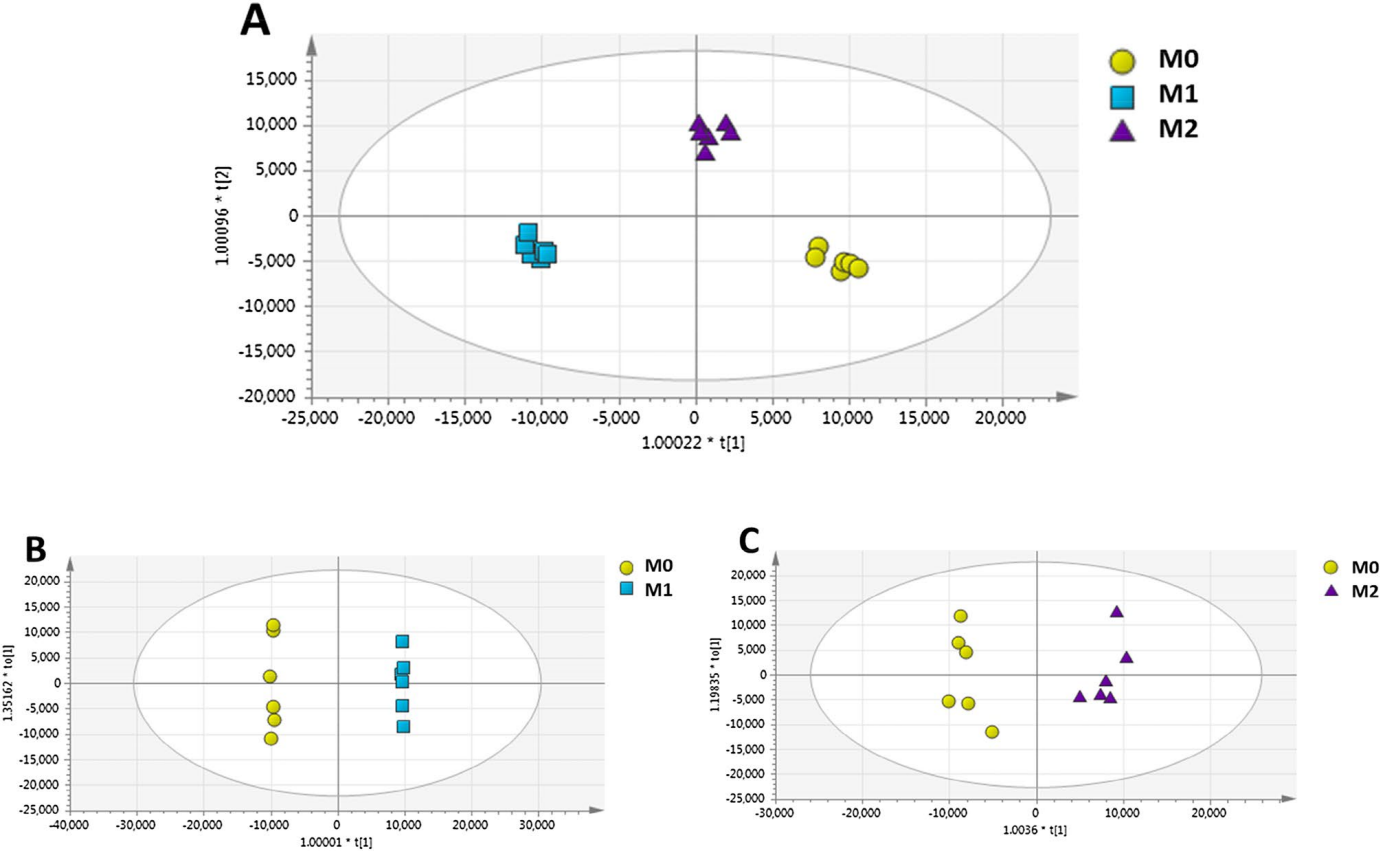
OPLS-DA scores plots of macrophage extracts after polarisation toward M1 and M2 states and their corresponding unpolarised (M0) controls. **a** M1 and M2 macrophage samples and their respective unpolarised controls (R^2^ = 0.647 and Q^2 =^ 0.847). **b** Two-way orthogonal comparison between M0 and M1 (R^2^ = 0.664 and Q^2^ = 0.899). **c** Two-way orthogonal comparison between M0 and M2 (R^2^ = 0.711 and Q^2^ = 0.838). M0 (yellow circles), M1 (blue squares), and M2 (purple triangles). n = 6

**Fig. 3:**
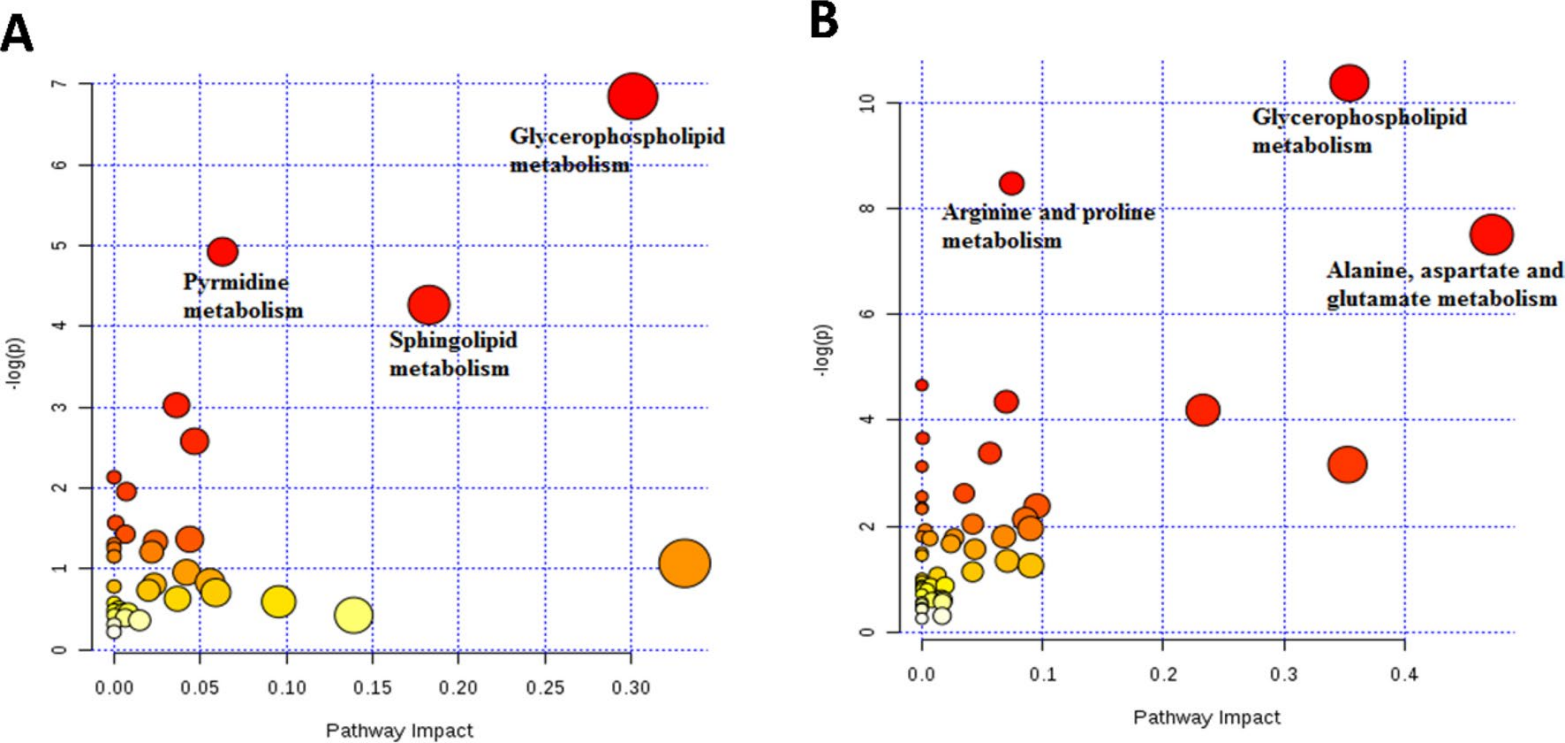
Summary of pathway analysis by MetaboAnalyst. The top-pathways are ranked by the gamma-adjusted p values for permutation per pathway (y-axis) and the total number of hits per pathway (x-axis). The colour graduated from white to yellow, orange and red as the values of both x and y increase. **a** Metabolic pathways significantly changed in M1 macrophages. **b** Metabolic pathways significantly changed in M2 macrophages

As previously mentioned, macrophages adopt a range of phenotypes in response to different micro-environmental stimuli. To generate M1 cells, naïve THP-1 macrophages were treated with GM-CSF, LPS (a potent endotoxin) and a Th1 cytokine, IFN-γ, while M2 cells were generated using M-CSF and a Th2 cytokine, IL4. These biomolecules have been shown to drive macrophage polarisation down the pro-inflammatory or anti-inflammatory path, respectively (Gordon and Plüddemann 2017; Mantovani et al. 2004). To confirm the efficiency of the polarisation approach used in this study, the morphology, surface phenotypic markers and cytokine profile of cells in response to different cytokine treatments were analysed. For the visualisation of cell cytoskeleton, different macrophage subsets were stained with fluorescently labelled phalloidin. M1 cells showed a less rounded and elongated morphology while M2 cells appeared rounded and stretched, displaying a ‘fried egg like’ morphology; M0 cells had rounded but less stretched cells (Supplementary 1 Fig. 1). Results obtained were in line with existing morphological data for different macrophage subsets (Rostam et al. 2016; Toniolo et al. 2015). To find out the surface phenotype of each cell population, immunofluorescence staining for calprotectin and mannose receptor (Huang et al*.*), which have been associated with M1 and M2 cells, respectively (Rostam et al. 2016), was carried out. As shown in Supplementary 1 Fig. 2, M1 cells expressed higher levels of calprotectin and lower levels of MR compared to M2 and M0 cells. On the other hand, M2 cells expressed the highest level of MR with no calprotectin expression. Additionally, the levels of cytokines in the supernatants collected from the macrophage subsets were analysed by enzyme-linked immunosorbent assay (Toniolo et al. 2015). M1 cells expressed significantly higher levels of pro-inflammatory cytokines, TNF-α and IL-1β compared to M2 and M0 macrophages (Supplementary 1 Fig. 3). As anticipated, our polarisation approach induced distinct M1 and M2 macrophages, characterised by increased expression of pro-inflammatory (calprotectin, TNF- α, IL-1 β) or anti-inflammatory markers (Huang et al. 2014), respectively.

#### Untargeted metabolomics analysis of THP-1 polarised macrophages

In order to explore the changes in the metabolic profile of THP-1 macrophages upon polarisation to M1 and M2 subsets and identify potential key characteristic metabolites for each macrophages polarisation state, the extracted intracellular metabolites from M0 group (unpolarised) and polarised groups were analysed using a LC–MS-based technique. 5474 features were detected in positive and negative modes with total of 644 metabolites were identified putatively (Supplementary 2). Figure 1 shows the chemical composition of THP-1 macrophages with the biological classifications of the identified metabolites. The metabolome was mainly constituted of glycerophospholipids (20%) and amino acids (17%), respectively followed by fatty acyls (7%), other lipids (6%), carbohydrates (5%), nucleotides (3%), sphingolipids (3%), co-factors and vitamins (2%), and energy metabolites (1%).

#### Feature selection and identification of potential biomarker metabolites

In order to reveal the key metabolites representing potential key characteristic metabolites for the two polarisation states of macrophages, the pre-processed data were imported to SIMCA-P to perform multivariate analysis. OPLS-DA was performed to distinguish M0, M1 and M2 groups. OPLS-DA scores plot showed tight clustering of the six replicates within each group and clear separation between the three different groups (M0, M1, and M2 macrophages) with R^2^ and Q^2^ values of 0.647 and 0.847, respectively (Fig. 2). Six replicates were deemed to be valid for this type of cell line-based studies (Alazzo et al. 2019; Surrati et al. 2016). This separation indicated a distinctive metabolic signature of M1, M2 and M0 macrophages.

Two-way comparison illustrated in Fig. 2b and c was performed to determine the mass ions which contributed to the separation between M0 and M1 or M2 macrophages based on their VIP values. The mass ions with VIP values greater than one were considered as potential key mass ions. OPLS-DA scores plot showed a clear separation between M0 and M1groups (Fig. 2b) with R^2^ and Q^2^ values of 0.664 and 0.899, respectively, demonstrating an acceptable OPLS-DA model (Eriksson et al. 2006). In parallel, univariate t-test with FDR correction was performed to identify significantly changed mass ions (FDR < 0.05) in M1 compared to the corresponding control (M0). Combination of multivariate and univariate analysis revealed 90 significantly changed metabolites in M1 macrophages (Supplementary 3). Similarly, in Fig. 2c, OPLS-DA scores plot showed clear separation between M0 and M2 macrophages with Q^2^ and R^2^ values of 0.838 and 0.711, respectively.

Again, univariate t-test with FDR correction was performed in parallel to identify significant mass ions changed in M2 group compared to the corresponding control (M0), revealing 49 significantly changed metabolites in M2 macrophages (Supplementary 4). Table 1 shows the top twenty significant metabolites with higher fold changes (1.97 to 15.44) in M1 and M2.

**Table 1:** The significant metabolites in THP-1 macrophages upon polarisation into M1 and M2

Putative metabolite	Exact mass	RT (min)	Formula	PubChem ID	ID confidence	Polarization state	Fold change	FDR
5-Hydroxy-l-tryptophan	220.0847	9.21	C_11_H_12_N_2_O_3_	439280	L3	M1	15.44	4.12E−06
Cer (d18:1/22:1)	619.5899	5.03	C_40_H_77_NO_3_	53481052	L3	M1	4.77	0.0002
Cytidine	243.0854	11.26	C_9_H_13_N_3_O_5_	6175	L2	M1	3.74	0.0029
C14 Ceramide	509.4806	5.12	C_32_H_63_NO_3_	5282310	L3	M1	3.45	6.36E−05
Ceramide (d18:1/16:0)	537.5120	5.10	C_34_H_67_NO_3_	5283564	L3	M1	3.96	0.0012
Ceramide (d18:1/18:0)	565.5435	5.10	C_36_H_71_NO_3_	53481047	L3	M1	4.73	0.0156
Ceramide (d18:1/22:0)	621.6068	5.10	C_40_H_79_NO_3_	53481048	L3	M1	6.84	0.0122
Ceramide (d18:1/24:0)	649.6385	5.10	C_42_H_83_NO_3_	5283576	L3	M1	5.76	0.0128
3-(4-hydroxyphenyl) pyruvate	180.0423	8.52	C_9_H_8_O_4_	5318321	L3	M2	2.19	0.0024
3-Dehydroxycarnitine	145.1103	6.15	C_7_H_15_NO_2_	725	L3	M2	3.47	0.0078
4-Imidazolone-5-propanoate	156.0534	9.76	C_6_H_8_N_2_O_3_	128	L3	M2	2.08	6.3E−05
Alpha-CEHC	278.1517	4.65	C_16_H_22_O_4_	9943542	L3	M2	2.67	6.3E−05
Ala-Ala	160.0847	8.59	C_6_H_12_N_2_O_3_	5460362	L3	M2	1.94	0.0023
*N*-Ac-l-Asp	175.0480	11.16	C_6_H_9_NO_5_	65065	L3	M2	1.07	0.0065
*N*-Ac-l-Glu	189.0636	10.85	C_7_H_11_NO_5_	185	L2	M2	3.57	0.0046
PC(O-14:0/16:0)	691.5514	5.27	C_38_H_78_NO_7_P	24779275	L3	M2	2.12	0.0076
PC(P-20:0/0:0)	535.3997	5.66	C_28_H_58_NO_6_P	52924063	L3	M2	3.99	0.0139
*N*-Acetylserotonin	218.1055	6.34	C_12_H_14_N_2_O_2_	903	L3	M1/M2	6.78/3.04	0.001/0.017

(+) indicates the metabolite increase upon polarisation. M1 and M2 indicate the polarisation states. ID confidence represents the level of metabolite identification based on (Schymanski et al. 2014; Sumner et al. 2014, 2007). *Ala-AlaD*-Alanyl-l-Alanine, *N-Ac-L-AspN*-acetyl-l-aspartate, *N-Ac-L-GluN*-acetyl-l-glutamate

### Pathway analysis

**Fig. 4:**
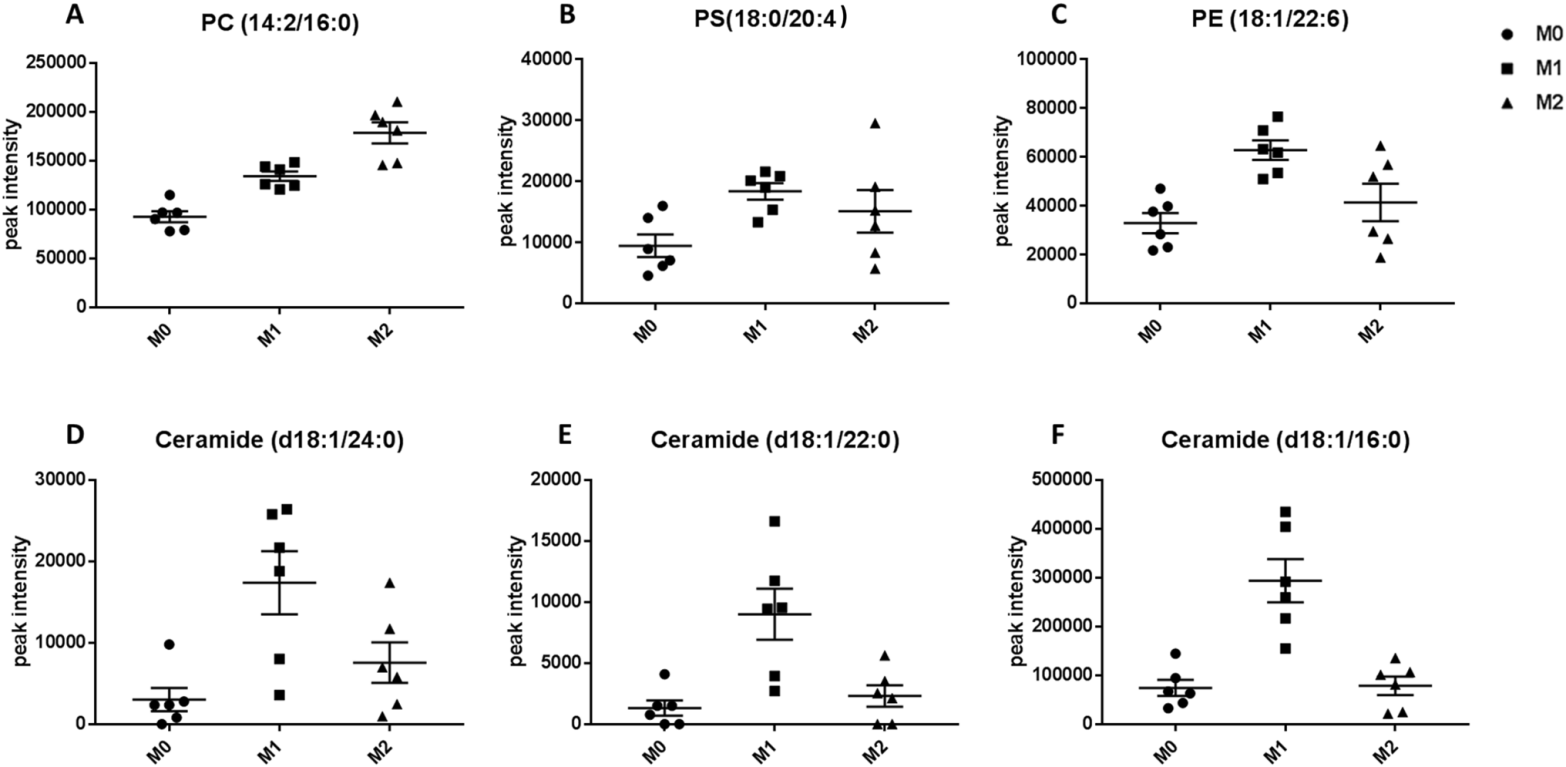
GLs and sphingolipids that significantly affected upon polarisation. **a** PC (14:2/16:0), **b** PS (18:0/20:4), and **c** PE (18:1/22:6) represent some GLs increased significantly in M1 or M2 macrophages compared to M0 macrophages. **d** Ceramide (d18:1/24:0), **e** Ceramide (d18:1/22:0) and **f** Ceramide (d18:1/16:0) as potential key characteristic metabolites of M1 macrophages and their significant increase compared to M0 and M2 macrophages

For further investigation to identify the metabolic pathways significantly altered by M1 and M2 polarisation states, a summary of pathway analysis is illustrated in Fig. 3a and b. Figure 3a and b show that glycerophospholipids (GLs) metabolism was significantly changed in M1 as well as M2. Glycerophospholipids constitute the main components of the cell membrane (Strickland et al. 1963). They mainly include phosphatidylcholines (PC), phosphatidylethanolamines (PE), phosphatidylserines (Strickland et al. 1963), phosphatidylinositols (PI) and phosphatidylglycerols (PG). The level of GLs in polarised macrophages (M1 and M2) was significantly increased and this results clearly from the increased levels of PC, PS and PE (Supplementary 3 and 4). Figure 4a–c show different GLs such as PC (14:2/16:0), PS (18:0/20:4), and PE (18:1/22:6) that significantly increased in M1 or M2. This agrees with recent research where Zhang et al. detected increase in the composition of medium- and long-chain polyunsaturated GLs, such as PS, PC, PE, and PI upon macrophages polarisation into M1 and M2 phenotypes, compared to M0 (Zhang et al. 2017). Generally, lipid metabolism contributes to the pro- or anti-inflammatory functions of macrophages by meeting energetic requirements and modulating membrane fluidity (Mukundan et al. 2009). However, GLs are also involved in multiple biological processes, such as inflammation and cell differentiation (Masoodi et al. 2015).

Sphingolipids and pyrimidine metabolisms were significantly altered in M1 macrophages as shown in Fig. 3a. Sphingolipids constitute a large class of bioactive and structural lipids representing the main lipid component of the eukaryotic plasma membrane. Sphingolipids are metabolised by a family of enzymes that regulate their metabolic interconnected network (Kihara et al. 2007). In recent years, different aspects of sphingolipids function have been investigated, including their role in inflammation (El Alwani et al. 2006), signalling pathways and metabolism (Zheng et al. 2006). The key metabolite in sphingolipids metabolism is ceramide which is generated either through another sphingolipid metabolism or in a de novo pathway at the endoplasmic reticulum (ER) (Siow and Wattenberg 2014). This obvious alteration in sphingolipid pathway is attributed to significant increase in the levels of some of sphingolipids and ceramides which could be considered as potential key characteristic metabolites for M1 macrophages. The levels of sphingolipids such as, ceramide (d18:1/24:0), ceramide (d18:1/22:0) and ceramide (d18:1/16:0) increased significantly in M1 macrophages as shown in Fig. 4a–c, respectively. It was demonstrated that stimulation of macrophages (RAW264.7) with LPS or another TLR-4 agonist elevates ceramide levels by multiple mechanisms, including de novo synthesis pathway (Sims et al. 2010b). Similarly, Cuschieri et al. reported that formation of ceramide could be involved in the gathering of Toll-like receptor (TLR) as a response to bacterial antigens such as lipopolysaccharide (LPS) (Cuschieri et al. 2007). Gathering and activation of TLR 4 in response to LPS drives a switch from oxidative phosphorylation into glycolysis which increases the reactive oxygen species (ROS) production (Kelly and O'Neill 2015), although our data showed no significant changes in the glycolysis intermediates. ROS is important for killing bacterial cells, which is significant function of M1 macrophages (Chanput et al. 2013). Others showed an increase in ceramide levels and inducing TLR4 mediated response in both leukaemia and epithelial cells in response to microbial ligands such as LPS (Cuschieri et al. 2006). Furthermore, a recent lipodomic study demonstrated an increase in the total cellular sphingolipids after TLR4 activation of murine macrophages (RAW 264.7 cells). Therefore, our results are in line with previous studies showing increase in ceramide production in pro-inflammatory macrophages. However, the mechanism responsible for increase in the synthesis of sphingolipids in TLR4-stimulated cells is still unknown (Sims et al. 2010a).

However, while the HILIC-MS-based approach employed in this study allowed the screening of a wide range of lipids and was thus ideally suited to initial determination of significant changes in lipid metabolism caused by macrophage polarisation states, the method was not suitable for separation and robust identification of individual lipids. Therefore, more detailed lipidomic approach using suitable separation techniques and structural confirmation based on fragmentation information will be another prospective approach revealing the lipid signature of M1 and M2 macrophages. This will be a complementary aspect with existing metabolomics data providing a clearer picture of macrophage responses.

**Fig. 5:**
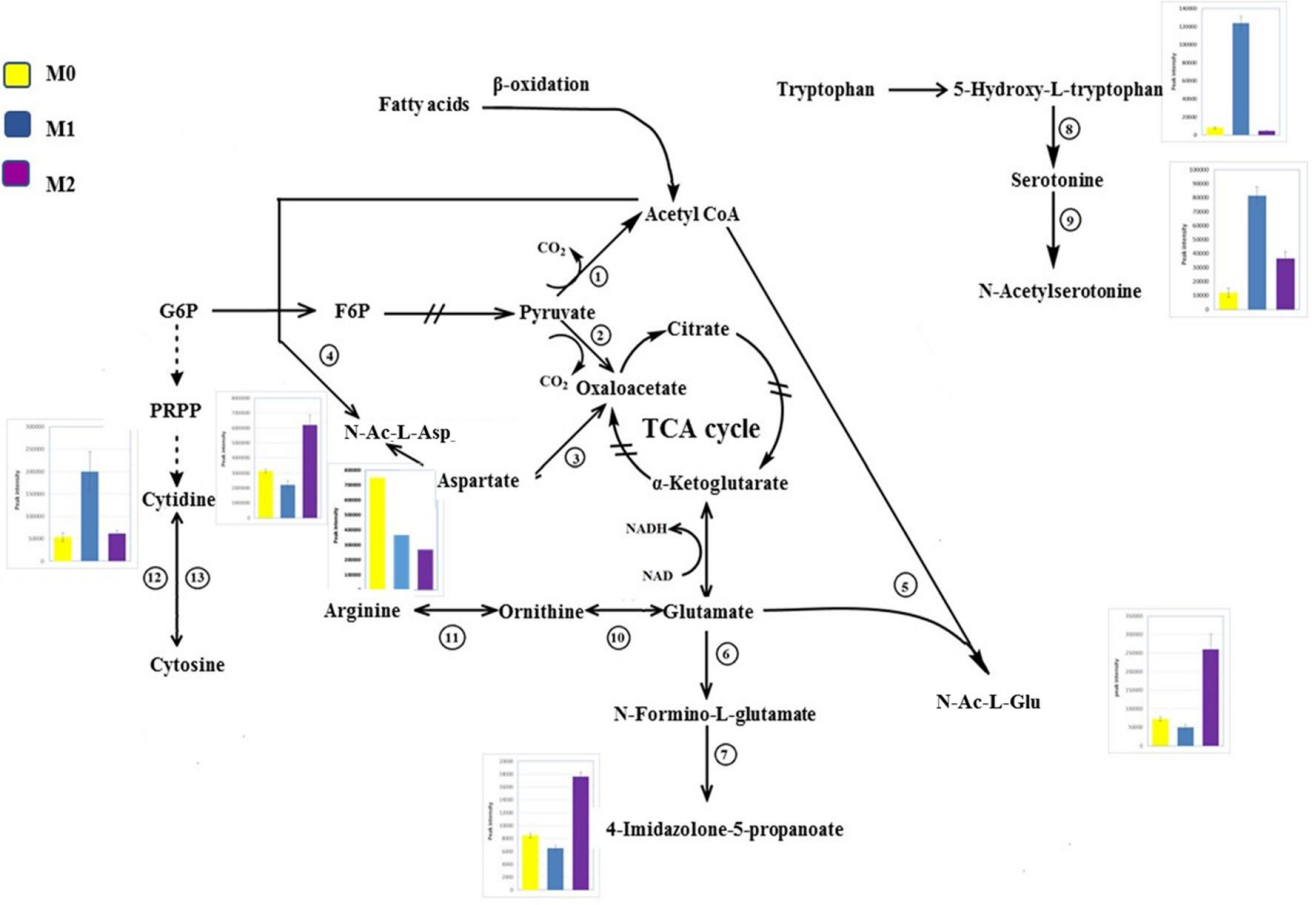
Significant metabolic pathways upon polarisation of THP-1 macrophages toward M1 and M2 states with bar graphs of key metabolites that significantly changed, the other metabolites either identified with no significant change (pyruvate, citrate, G6P, glutamate, ornithine and arginine) or unidentified. Metabolic enzymes are as follows; 1: pyruvate dehydrogenase, 2: pyruvate carboxylase, 3: glutamate-aspartate aminotransferase, 4: l-aspartate *N*-acetyltransferase, 5: *N*-acetylglutamate synthetase, 6: formiminoglutamase, 7: imidazolonepropionase, 8: tryptophan 5-monooxygenase, 9: arylalkylamine *N*-acetyltransferase, 10: ornithine aminotransferase, 11: arginase-1, 12: ribosylpyrimidine nucleosidase, 13: pyrimidine-nucleoside phosphorylase. G6P: glucose 6-phosphate, PRPP: 5-phosphoribosyl 1-pyrophosphate, F6P: fructose 6-phosphate. The bar charts show the peak intensity (y-axis) of corresponding metabolites, from the top to the bottom and left to right (cytidine, *N*-Ac-l-Asp, *N*-Ac-l-Glu, 4-imidazolone-5-propanoate, 5-hydroxy-tryptophan and *N*-acetylserotonine). M0 in yellow, M1 in blue and M2 in purple

Pyrimidine metabolism was another significantly perturbed pathway in M1 macrophages as can be seen in Fig. 3a. Pyrimidine is found in the form of nucleic acid and vitamins in living systems. It represents the basic nucleus in RNA and DNA. Therefore, it has been found to be involved in several biological activities (Bhat and Kumar 2017). Biologically, pyrimidines are considered as a very important class and represent the most abundant member of diazine family with uracil and thymine (Sharma et al. 2014). Numerous studies in the literature showed a broad range of therapeutic activity of pyrimidine nucleus-containing compounds such as antibacterial (Nargund et al. 1992) and antitubercular (Sawant and Kawade, 2011). Our results showed significant increase in the levels of cytidine, a pyrimidine that can be used as a potential new key characteristic metabolite for M1 macrophages (Fig. 5). These metabolites are involved in pyrimidine metabolism (KEGG map 00240). Increase in the levels of pyrimidines could result from pentose phosphate pathway (PPP) (KEGG map 00030) which is well known to be induced in M1 macrophages, and it enhances the production of pyrimidines as previously reported (Galván-Peña and O’Neill 2014). Nevertheless, metabolites involved in PPP showed no significant change in our results unexpectedly. Intensive research has been carried out to investigate activity of pyrimidines in inflammation. Bonnert et al. reported remarkable activity of pyrimidine derivatives against inflammation and immune disorders such as rheumatoid arthritis, atherosclerosis, and Crohn's disease (Bonnert et al. 2003). Furthermore, Nargund et al. showed potent antibacterial activity of pyrimidine derivatives against various gram-negative and gram-positive bacteria (Nargund et al. 1992). Collectively, these studies have revealed the activity of pyrimidines in inflammation and bacterial attack which is a pivotal role for M1 macrophages (Chanput et al. 2013). Also, purines along with pyrimidines are key components in the production of RNA and DNA. They have crucial role in energy system (e.g., ATP) (Jankowski et al. 2005) and enhanced PPP flux increases the production of purines as well as pyrimidines (Bedard and Krause 2007). Our data demonstrated a significant increase in the purines; hypoxanthine and inosine (Supplementary 3). In agreement with our results, hypoxanthine was reported to be increased in the presence of LPS (Rattigan et al. 2018). Pyrimidines and purines can be used for the activated cell biosynthesis and provide NADPH which is the substrate for NADPH oxidase in order to produce reactive oxygen species (Bedard and Krause 2007) that act as a bacterial killing mechanism (West et al. 2011) representing the main function of M1 macrophages. Furthermore, a previous study showed that the purines analogue (6-*N*-hydroxylaminopurine) has antibacterial and antiviral activity (Krajewski et al. 2017).

Figure 5 shows two metabolites with significant increase that could also be considered as potential key characteristic metabolites for M1 macrophages; 5-hydroxy-l-tryptophan and *N*-acetylserotonin which are intermediates in tryptophan metabolism (KEGG map 00380). 5-hydroxy-l-tryptophan are generated as a result of tryptophan breakdown by tryptophan hydroxylase (KEGG R01814) followed by the reaction KEGG R02701 producing serotonin. *N*-Acetylserotonin is produced from serotonin by the reaction KEGG R02911. The precursor tryptophan is an amino acid required for metabolic functions and protein synthesis. It cannot be synthesised in the body; therefore, it must be supplied in the form of proteins. Although many studies have focused on the role of tryptophan metabolism in the last decades, its role and the role of intermediates in the immune system are not yet fully understood. However, tryptophan metabolism and inflammatory responses are thought to be associated with several diseases and pathological conditions (Moffett and Namboodiri 2003). For instance, Tarique et al. revealed a role of 5-hydroxy tryptophan in innate immunity as it attracts mast cells to the inflammation site (Kushnir-Sukhov et al. 2006). Moreover, Nakumura et al. showed that 5-hydroxy tryptophan enhanced phagocytosis in murine macrophages (Nakamura et al. 2008), a key property by which M1 macrophages eliminate foreign particles (Tarique et al. 2015).

On the other hand, arginine, proline metabolism, and alanine, aspartate and glutamate metabolism were all significantly altered in M2 macrophages (Fig. 3b). l-Arginine is considered as a non-essential amino acid at the whole organism level, but it is important to be supplemented in certain diseases (Bansal and Ochoa 2003). It is believed that mammalian arginine metabolism and its cellular regulation is a complex process (Morris 2006). l-Arginine is produced from de novo arginine production or cellular protein break-down. Many amino acids are involved in the biosynthesis of arginine such as l-citrulline, l-ornithine and l-glutamine. Furthermore, l-arginine represents a precursor for the synthesis of urea, nitric oxide, l-glutamate, polyamines, l-proline, and creatine (Wu and Morris 1998). Corraliza et al. previously demonstrated a key aspect of arginine metabolism which showed that different enzymes involved in arginine metabolism are promoted in macrophages based on their polarisation state (M1 or M2). Arginine in macrophages is catabolised by either iNOS in M1 macrophages producing NO and citrulline or arginase-1 in M2 macrophages producing l-ornithine, polyamines (putrescine, spermidine and spermine), and urea, which are involved critically in wound healing and tissue repair function of M2 macrophages (Corraliza et al. 1995). In agreement with this statement our results showed significant increase in l-citrulline (Supplementary 3). This has been proved by Sugimoto et al. demonstrating excessive increase in l-citrulline in LPS-activated macrophages (Sugimoto et al. 2012). Figure 5 shows three of potential key characteristic metabolites for M2 macrophages; *N*-acetyl-l-glutamate (*N*-Ac-l-Glu), *N*-acetyl-l-aspartate (*N*-Ac-l-Asp), and 4-imidazolone-5-propanoate that significantly increased in these cells. *N*-acetyl-l-glutamate is produced by a reaction of acetyl-CoA with l-glutamate (KEGG R00259). Acetyl-CoA is generated from β-oxidation of fatty acids which was proved to be utilised in M2 macrophages (Huang et al. 2014). However, l-glutamate is interconverted from l-ornithine by the enzyme ornithine aminotransferase (KEGG R00667). As aforementioned, l-ornithine is known to be produced in M2 macrophages from l-arginine via arginase-1, and is represented as a substrate for the enzyme ornithine decarboxylase in the synthesis of polyamines induced in M2 macrophages (Rath et al. 2014). The other significantly altered pathway in M2 macrophages was alanine, aspartate, and glutamate metabolism, which is connected to TCA cycle. No significant change was shown in TCA cycle intermediates, which could indicate intact and functioning TCA cycle tightly linked to fully functioning oxidative phosphorylation (OXPHOS) of mitochondria. OXPHOS was reported as a hallmark of M2 macrophages (Galván-Peña and O’Neill 2014). Therefore, l-aspartate could be driven from TCA cycle by oxaloacetate (KEGG R00355) and consequently to be consumed in a reaction with acetyl-CoA to produce *N*-Ac-l-Asp by l-aspartate *N*-acetyltransferase (KEGG R487) which could explain the decrease in the level of l-aspartate and increase in the level of *N*-acetyl-l-aspartate. 4-Imidazolone-5-propanoate could represent another source for glutamate (KEGG R02288 and R02285) which, in addition to producing *N*-Ac-l-Glu, glutamate could feed into TCA cycle by being converted to α-ketoglutarte by glutamate dehydrogenase.

Surprisingly, glycolysis metabolic pathway, PPP and OXPHOS showed no significant difference in their intermediates although many of these metabolites were identified (Supplementary 4). These metabolic pathways represent hallmarks for M1 (glycolysis and PPP) and M2 macrophages (OXPHOS which connected to TCA cycle) as discussed earlier. These results can be owed to different reasons. Firstly, macrophages heterogenicity and diversity enable the generation of infinite number of phenotypes with different profiles under the impact of varying stimuli (Martinez et al. 2013; Weigert et al. 2018). Our protocol of polarisation used multi stimuli for M1 and M2 macrophages which were LPS, GM-CSF and IFN-γ for M1 macrophages and IL-4 and M-CSF for M2 macrophages. However, most of the studies used LPS or/and IFN-γ for M1 and IL-4 for M2 macrophages. GM-CSF and M-CSF can give rise to pro-inflammatory and anti-inflammatory macrophages, respectively, and so it is expected that they impact the metabolic profile of macrophage phenotypes (Akagawa 2002; Rattigan et al. 2018). It was already reported that using LPS alone, IFN-γ alone or both of them together gives rise to different metabolic response of macrophages (Rattigan et al. 2018). Therefore, metabolic responses to different stimuli can be sufficiently different, contradictory and seriously complex. This in turn suggests that the studies with single or limited number of stimuli do not reflect the complexity of the picture in vivo (Rattigan et al. 2018). Secondly, and most importantly, most of the previous studies discussed the metabolic signature of M1 and M2 in mice macrophages, either primary or cell line stimulated with in vitro stimuli whereas our experiment was conducted using human cell line (THP-1 cells). In fact, macrophages from different species, residing different tissues or arising from different progenitors may show different biological and metabolic signature, even to defined stimuli (Artyomov et al. 2016; Weigert et al. 2018). This could highlight the need to focus on primary human macrophages in the future studies to acquire the more accurate reflection of the biology and signature of macrophages however the large number of cells required for conventional metabolomics studies and access to human tissue limit the feasibility of such studies.

## Conclusion

In this study, LC–MS-based metabolite profiling was used successfully to characterise M0, M1 and M2 macrophages. Furthermore, uni- and multivariate analysis demonstrated that this technique is sensitive enough to detect subtle changes in a wide range of metabolites upon polarisation of macrophages and identify the distinct metabolic signature of M1 and M2 phenotypes. Various metabolic pathways were significantly affected in these macrophage phenotypes. Given the significant number of diseases associated with each phenotype such as chronic inflammatory diseases or cancer progression, the identified key metabolites will improve the understanding of underlying mechanisms in macrophages polarisation. Moreover, this data provides new insight for the rational design of novel immune modulatory strategies such as targeting specific pathways associated with different macrophage subsets in inflammatory diseases. Future studies should take into consideration different type of stimuli for macrophage polarisation to resemble the multiple stimuli present in vivo to account for the diverse range of signalling pathways resulting in different immune-metabolic responses (Lachmandas et al. 2016). Furthermore, the origin of macrophages for investigation of metabolic signature of different macrophage subsets should be considered since it could represent essential biochemical information revealing key pathways in specific diseases.

## Electronic supplementary material

Below is the link to the electronic supplementary material.

Supplementary file1 (DOCX 905 kb)

Supplementary file2 (XLSX 12720 kb)

Supplementary file3 (XLSX 19 kb)

Supplementary file4 (XLSX 15 kb)
