# Supplementary material for: Metabolic characterisation of THP-1 macrophage polarisation using LC–MS-based metabolite profiling
Source: Metabolomics. 2020 Feb 29;16(3):33. doi: 10.1007/s11306-020-01656-4 (PMC7049298; doi:10.1007/s11306-020-01656-4)
Supplement: Supplementary file 1 — Supplementary file1 (DOCX 905 kb) [file 11306_2020_1656_MOESM1_ESM.docx]

**SUPPLEMENTARY IINFORMATION**

**Metabolic characterisation of THP-1 macrophage polarisation using LC-MS-based metabolite profiling**

Alaa Abuawad^1, 2^, Chidimma Mbadugha^3^, Amir M. Ghaemmaghami^3^, Dong-Hyun Kim^1*^

^1^Centre for Analytical Bioscience, Division of Advanced Materials and Healthcare Technologies, School of Pharmacy, University of Nottingham, UK

^2^Department of Pharmaceutical Sciences and Pharmaceutics, Faculty of Pharmacy, Applied Science Private University, Amman, Jordan.

^3^Division of Immunology, School of Life Sciences, Faculty of Medicine and Health Sciences, University of Nottingham, Nottingham, UK

*Correspondence to: Email: [dong-hyun.kim@nottingham.ac.uk](mailto:dong-hyun.kim@nottingham.ac.uk) Tel: +44 1157484697

**Characterization of macrophages polarization**

**DAPI**

**Phalloidin**


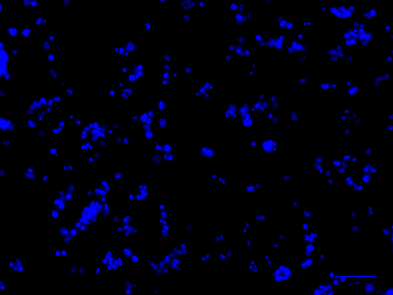

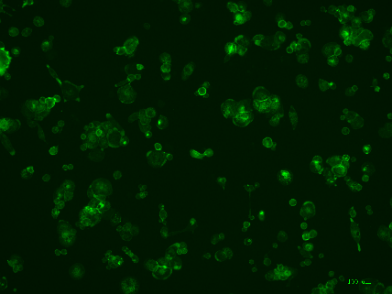


**M0**


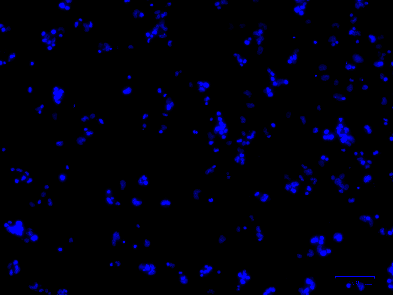

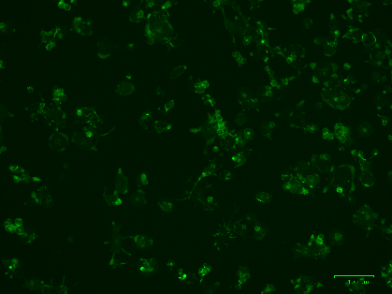


**M1 c10**


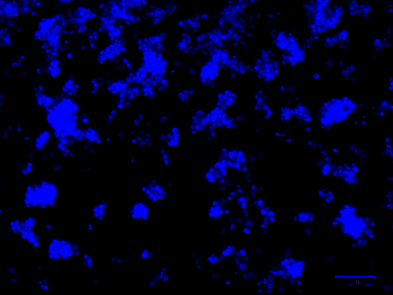

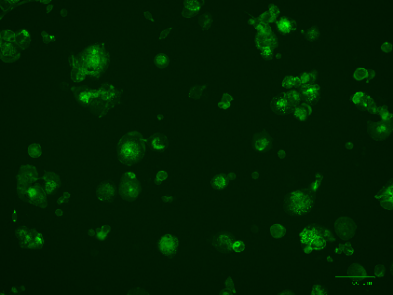


**M2 c10**

**Supplementary 1 Figure 1:** Morphology of cytokine polarised THP-1 cells. Cells were stained with phalloidin (green) and DAPI (blue) for the visualization of cytoskeleton. Images were taken at 10x magnification; scale bar = 100μm.


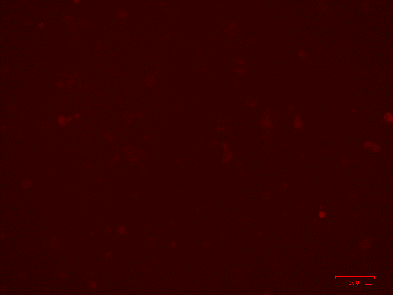

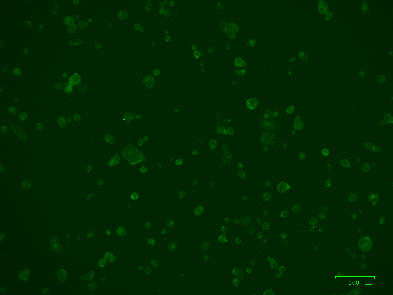


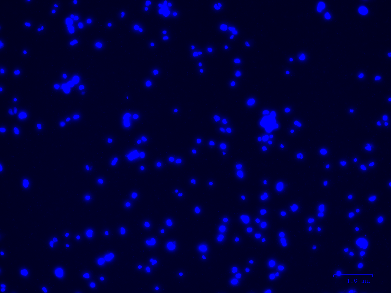


**M0**


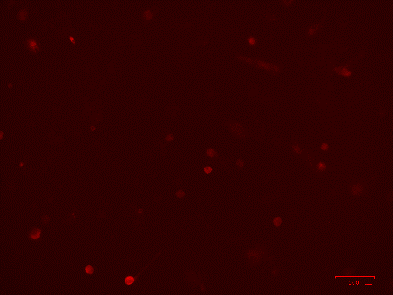

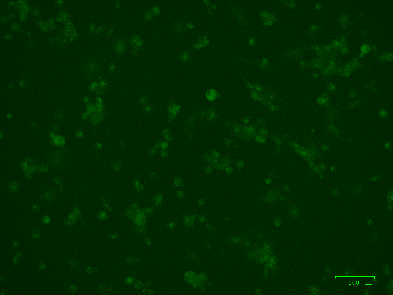

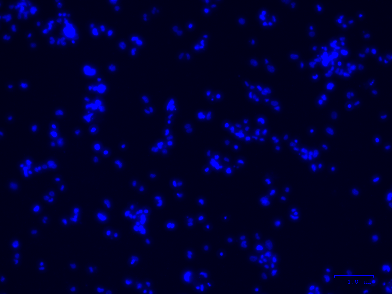


**M1**

**M2**


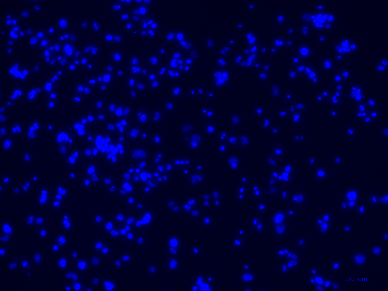

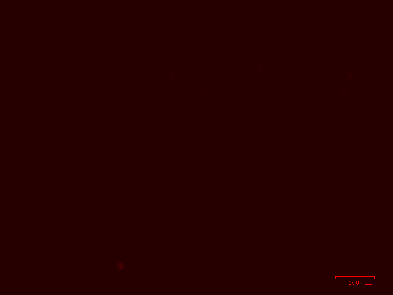

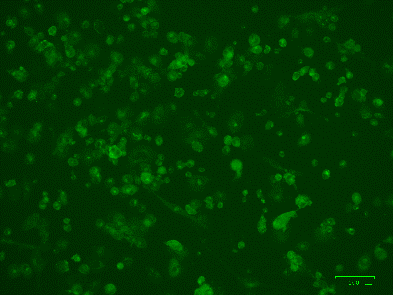


**B**

**Supplementary 1 Figure 2**: Expression of surface markers by cytokine polarized THP-1 macrophages. A) Immunofluorescent images of showing mannose receptor (green) and calprotectin (red) expression; nuclei were stained with DAPI (blue). B) Mean fluorescent intensities of M1, M2 and M0 cells.

**Supplementary 1 Figure 3**: Cytokine expression by macrophages. Following 6 h PMA activation, cells were treated with GM-CSF (50ng/ml), LPS (100 ng/mL) and IFN-γ (20 ng/mL) or M-CSF (50 ng/mL) and IL-4 (20 ng/mL) for up to 72 h to generate M1 or M2 polarised cells, respectively. A) TNF-α B) IL-1β expression. Data is presented as mean ± SD (n=3).
